# Supplementary material for: Heparan-6-O-Endosulfatase 2 Promotes Invasiveness of Head and Neck Squamous Carcinoma Cell Lines in Co-Cultures with Cancer-Associated Fibroblasts
Source: Cancers (Basel). 2023 Oct 27;15(21):5168. doi: 10.3390/cancers15215168 (PMC10650326; doi:10.3390/cancers15215168)
Supplement: Supplementary file 1 [file cancers-15-05168-s001.zip › cancers-2674756-supplementary.pdf]

Supporting information

Heparan-6-*O*-endosulfatase 2 promotes invasiveness of head and neck squamous carcinoma cell lines in co-cultures with cancer associated fibroblasts

Pritha Mukherjee[1*], Xin Zhou[1,2*], Julius Benicky[1,3], Aswini Panigrahi[1,3], Reem Aljuhani[1], Jian Liu[4], Laurie Ailles[5,6], Vitor H. Pomin[7,8], Zhangjie Wang[9], Radoslav Goldman[1,3,10]

[1] Department of Oncology, Lombardi Comprehensive Cancer Center, Georgetown University, Washington, DC 20057, USA
[2] Biotechnology Program, Northern Virginia Community College, Manassas, VA 20109
[3] Clinical and Translational Glycoscience Research Center, Georgetown University, Washington, DC 20057, USA
[4] Division of Chemical Biology and Medicinal Chemistry, Eshelman School of Pharmacy, University of North Carolina, Chapel Hill, NC 27599, USA
[5] Department of Medical Biophysics, University of Toronto, Toronto, ON M5G 1L7, Canada
[6] Princess Margaret Cancer Centre, University Health Network, Toronto, ON M5G 1L7, Canada
[7] Department of BioMolecular Sciences, University of Mississippi, Oxford, MS 38677, USA
[8] Research Institute of Pharmaceutical Sciences, School of Pharmacy, University of Mississippi, Oxford, MS 38677, USA
[9] Glycan Therapeutics, LLC, 617 Hutton Street, Raleigh, NC 27606
[10] Department of Biochemistry and Molecular & Cellular Biology, Georgetown University, Washington, DC 20057, USA

Correspondence: rg26@georgetown.edu; Tel: +1-202-687-9868
*Co-first Author

Content

Figure S1 Sulf2-KO of Cal33 cells using CRISPR/Cas9

Figure S2 Targeted Mass spectrometry for the detection of Sulf-2

Figure S3 Mono-culture spheroids of HNSCC-WT, Sulf-2-KO and HNCAF37 cells

Table S1 Inhibition of Sulf2-mediated desulfation of heparan sulfate by HfFucCS

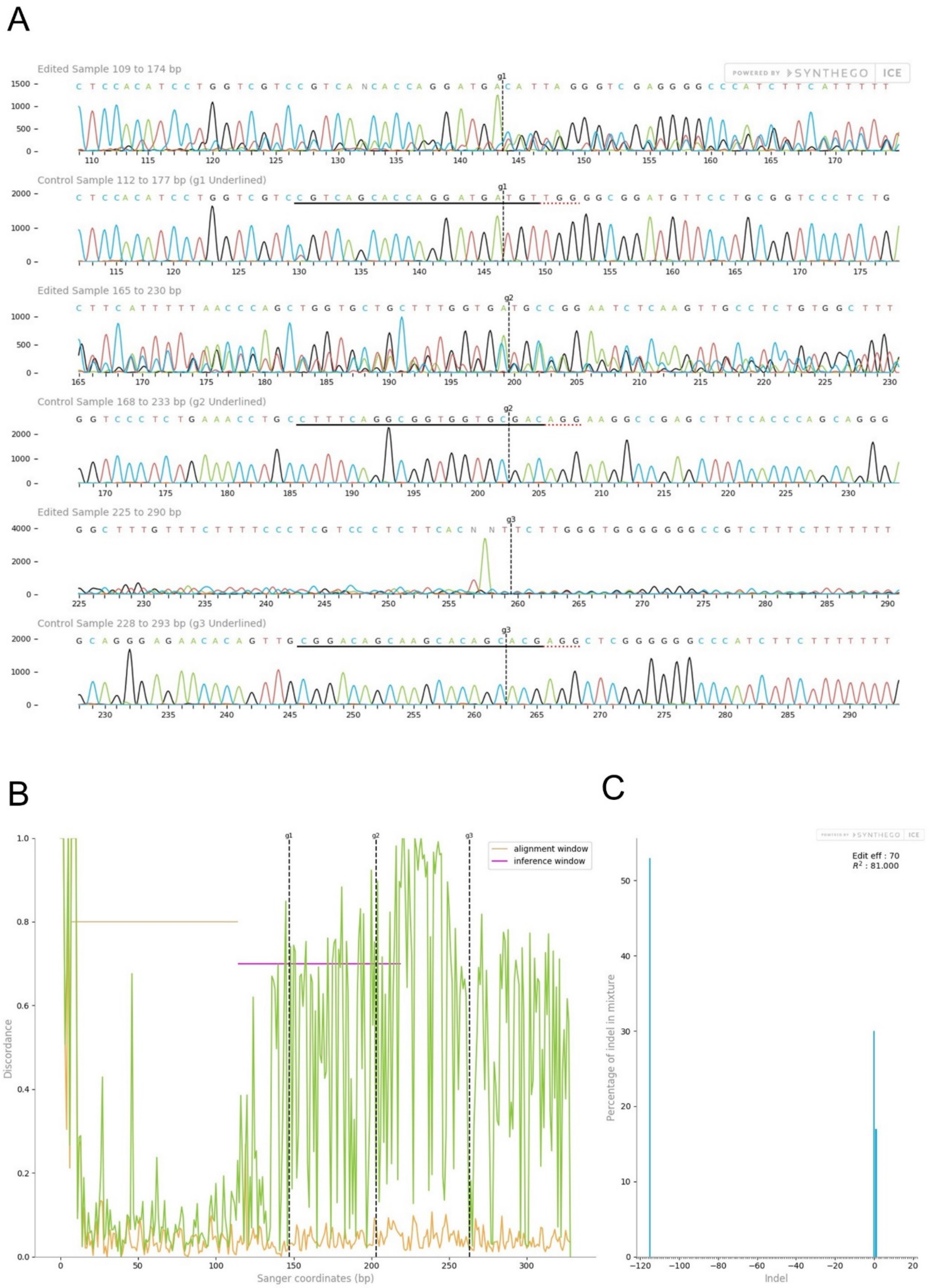

Figure S1. Inference of CRISP Edits (ICE) analysis of Sulf-2 knockout Cal33 cells. (A) Comparison of wild-type (bottom) and knockout (upper) trace files obtained by Sanger sequencing of the edited genomic DNA region. (B) Discordance plot showing the base-by-base level of disagreement between the non-edited wild-type (orange line) and the edited sample

(green line) in the inference window around the edited region. (C) Histogram showing the percentual distribution of indel sizes in the edited population. Three sgRNAs are referred to as g1, g2, and g3.

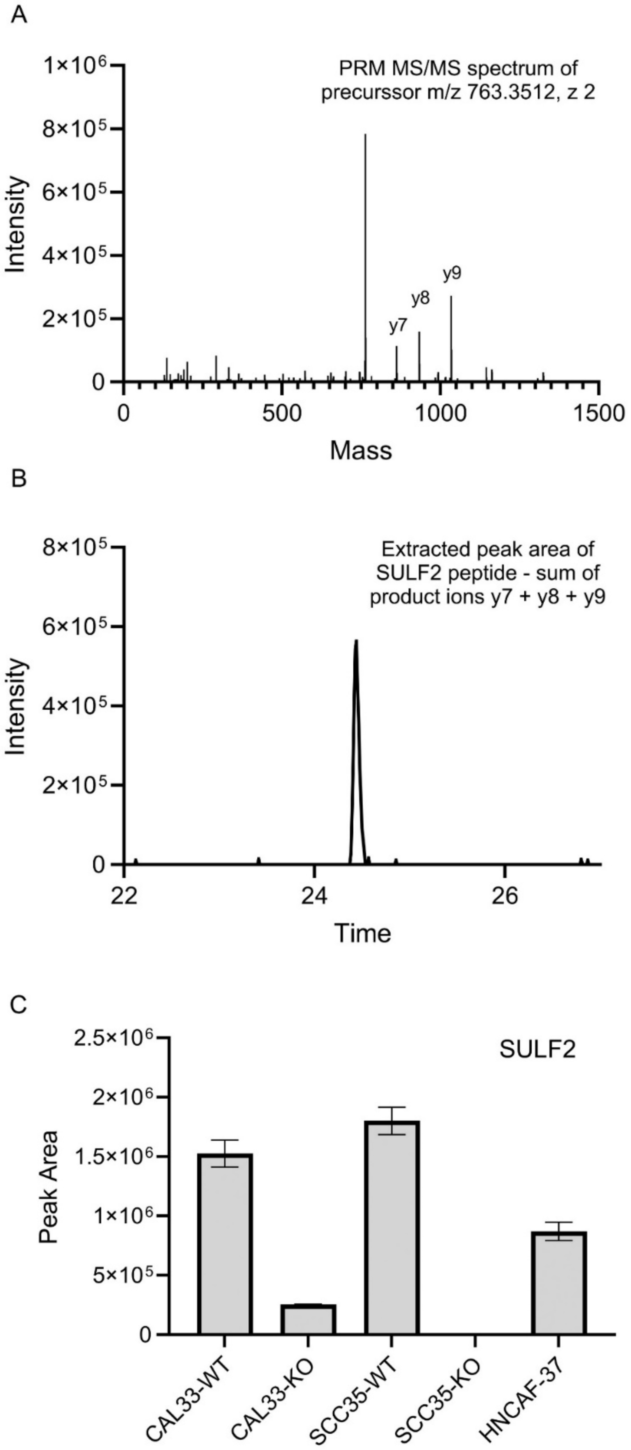

Figure S2. LC-MS/MS-PRM quantification of Sulf-2 in the secretomes of wild-type and CRISPR/Cas knockout cells. (A) Representative PRM spectrum of SCC35-WT sample; the matched Sulf-2 product ions y7 (862.41), y8 (933.44), and y9 (1034.49) are indicated. (B)

Extracted peak area of the Sulf-2 peptide using the sum of y7, y8 and y9 product ions at RT 24.44 min. (C) Relative concentration of Sulf-2 in SCC35, Cal33 and HNCAF37 media samples. WT, wild type cells, KO, Sulf-2-KO cells.

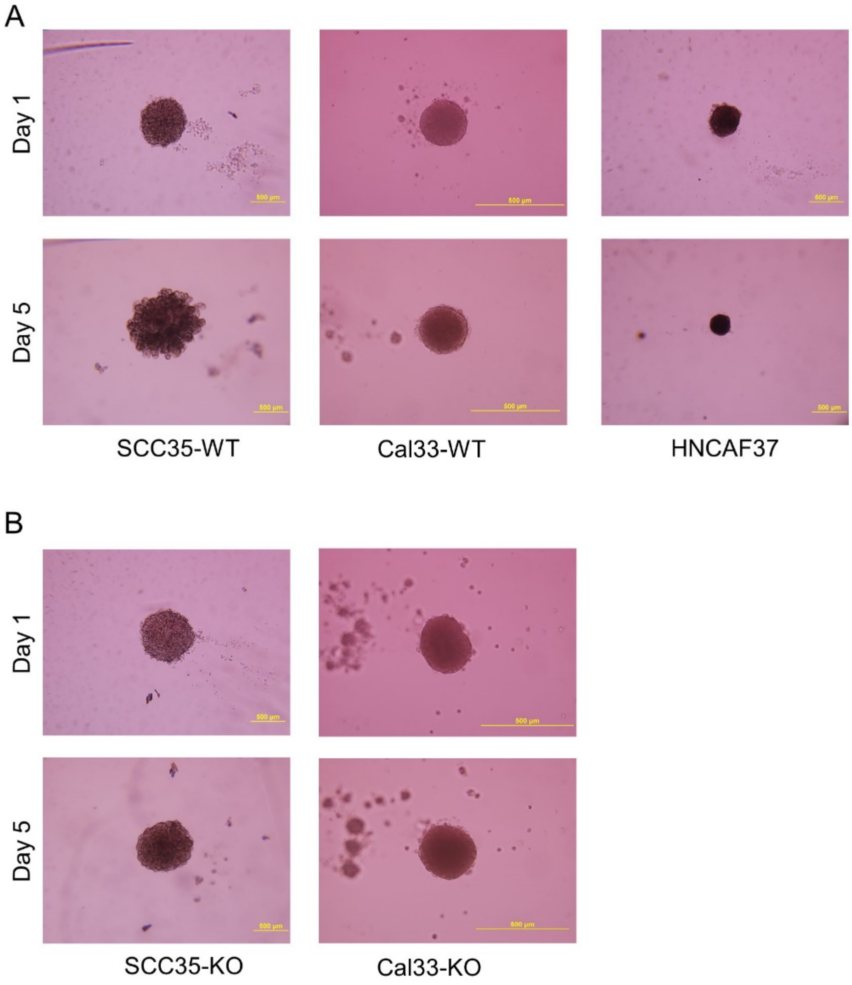

Figure S3. Mono-culture spheroids in Matrigel. (A) Representative images of spheroids of HNSCC cells SCC35 and Cal33 and HNCAF37 at day 1 and day 5. (B) Representative images of spheroids formed by SCC35-KO and Cal33-KO at day 1 and day 5. Scale bar 500 μm.

Table S1. Inhibition of Sulf-2-mediated desulfation of heparan sulfate by HfFucCS

Disaccharide	Molar percentage (%)		
	No treatment	Sulf2	Sulf2+HfFucCS
ΔUA2S-GlcNS6S	19.4	6.0	15.4
ΔUA2S-GlcNS	4.2	16.1	3.7
ΔUA-GlcNS6S	16.0	11.7	12.5
ΔUA-GlcNS	14.1	18.9	11.2
ΔUA2S-GlcNAc6S	1.9	0.9	1.5
ΔUA2S-GlcNAc	1.1	1.7	0.9
ΔUA-GlcNAc6S	7.3	7.7	5.7
ΔUA-GlcNAc	36.1	36.9	49.0

HS from porcine mucosa (25 μg) was desulfated with Sulf-2 (1 μg) with or without HfFucCS (5 μg) for 8 hrs as described in the Methods. Non-treated HS served as a control. The table shows the distribution of individual HS disaccharides as a molar percentage of the sum of all disaccharide forms. Trisulfated disaccharide ΔUA2S-GlcNS6S represents a major Sulf-2 substrate; its desulfation yields ΔUA2S-GlcNS product. Abbreviations: UA, uronic acid; GlcN, glucosamine; GlcNAc, N-acetylglucosamine; S, sulfate.
